# The Role of Epithelial–Mesenchymal Transition in Osteosarcoma Progression: From Biology to Therapy

**DOI:** 10.3390/diagnostics15050644

**Published:** 2025-03-06

**Authors:** Andrei-Valentin Patrașcu, Elena Țarcă, Ludmila Lozneanu, Carmen Ungureanu, Eugenia Moroșan, Diana-Elena Parteni, Alina Jehac, Jana Bernic, Elena Cojocaru

**Affiliations:** 1 Department of Morphofunctional Sciences I—Pathology, Faculty of Medicine, “Grigore T. Popa” University of Medicine and Pharmacy, 700115 Iasi, Romania; patrascu_andrei-valentin@d.umfiasi.ro (A.-V.P.); carmen.ungureanu@umfiasi.ro (C.U.); eugenia.morosan@umfiasi.ro (E.M.); parteni.diana-elena@d.umfiasi.ro (D.-E.P.); elena2.cojocaru@umfiasi.ro (E.C.); 2 Department of Surgery II—Pediatric Surgery, Faculty of Medicine, University of Medicine and Pharmacy “Gr. T. Popa”, 700115 Iasi, Romania; 3 Department of Morphofunctional Sciences I—Histology, Faculty of Medicine, “Grigore T. Popa” University of Medicine and Pharmacy, 700115 Iasi, Romania; ludmila.lozneanu@umfiasi.ro; 4 Second Dental Medicine Department, Faculty of Dental Medicine, “Grigore T. Popa” University of Medicine and Pharmacy, 700115 Iasi, Romania; alina.jehac@umfiasi.ro; 5 Discipline of Pediatric Surgery, “Nicolae Testemițanu” State University of Medicine and Pharmacy, MD-2001 Chisinau, Moldova; jana.bernic@usmf.md

**Keywords:** epithelial–mesenchymal transition, osteosarcoma, therapeutic target, malignant bone tumor, mesenchymal tumor

## Abstract

Osteosarcoma (OS) is the most common primary malignant bone tumor, predominantly affecting children, adolescents, and young adults. Epithelial–mesenchymal transition (EMT), a process in which epithelial cells lose their cell–cell adhesion and gain migratory and invasive properties, has been extensively studied in various carcinomas. However, its role in mesenchymal tumors like osteosarcoma remains less explored. EMT is increasingly recognized as a key factor in the progression of osteosarcoma, contributing to tumor invasion, metastasis, and resistance to chemotherapy. This narrative review aims to provide a comprehensive overview of the molecular mechanisms driving EMT in osteosarcoma, highlighting the involvement of signaling pathways such as TGF-β, transcription factors like Snail, Twist, and Zeb, and the role of microRNAs in modulating EMT. Furthermore, we discuss how EMT correlates with poor prognosis and therapy resistance in osteosarcoma patients, emphasizing the potential of targeting EMT for therapeutic intervention. Recent advancements in understanding EMT in osteosarcoma have opened new avenues for treatment, including EMT inhibitors and combination therapies aimed at overcoming drug resistance. By integrating biological insights with clinical implications, this review underscores the importance of EMT as a critical process in osteosarcoma progression and its potential as a therapeutic target.

## 1. Introduction

Epithelial–mesenchymal transition (EMT) is a biological process whereby epithelial cells lose their specific characteristics, such as tight adhesion to neighboring cells, polarity, and epithelial structure, and acquire a mesenchymal phenotype characterized by increased mobility, invasiveness, and the ability to migrate [1,2]. This process is regulated by a variety of molecular signals and transcription factors, including TGF-β, Wnt, and Notch, and is associated with changes in the expression of cell adhesion proteins, such as cadherins, as well as the activation of migration-associated proteins, such as fibronectin and vimentin [3]. EMT plays a crucial role in various biological processes, including embryonic development, wound healing, and tumor progression [4]. In the context of cancer, EMT contributes to tumor invasion, metastasis, and treatment resistance, and is involved in the progression of many types of tumors, including osteosarcoma [5,6].

### Osteosarcoma: Clinical and Molecular Characteristics

Osteosarcoma (OS) is a highly aggressive malignancy with complex clinical and molecular characteristics that significantly affect its diagnosis, treatment, and prognosis. Epidemiological studies stated that OS is the most common primary malignant bone tumor, primarily affecting children, adolescents, and young adults, with a peak incidence during periods of rapid skeletal growth, typically between the ages of 10 and 24 [7]. A second, smaller peak of OS incidence is observed in older adults, typically over the age of 60, often associated with pre-existing conditions such as Paget’s disease of bone, bone infarcts, or previous radiation exposure [7,8]. It typically arises in the metaphyseal regions of long bones, particularly around the knee, including the distal femur and proximal tibia, as well as in the proximal humerus. The incidence of this malignancy is slightly higher in males than in females [9].

Patients often present with localized pain and swelling around the affected bone, which may be associated with increased activity or trauma. Other symptoms may include limited range of motion and, in advanced cases, systemic signs such as fever or weight loss [10,11]. Imaging studies, including X-rays, CT scans, and MRIs, reveal characteristic features such as bone destruction, soft tissue masses, and the presence of periosteal reactions (e.g., Codman’s triangle). The classic “sunburst” pattern or “onion skin” appearance may also be observed [10].

Osteosarcoma prognosis depends largely on tumor size, location, and metastasis at diagnosis [12]. Patients with localized disease have a 5-year survival rate of 60–70%, but this drops to 20–30% for those with metastatic spread, especially to the lungs. Chemotherapy response also plays a critical role in determining outcomes [13].

Treatment typically involves a combination of surgery, chemotherapy and radiotherapy. Limb-salvage surgery is preferred when possible, with amputation being less common today. Standard chemotherapy regimens include methotrexate, doxorubicin, and cisplatin (MAP). Neoadjuvant chemotherapy is used to shrink the tumor before surgery, followed by adjuvant chemotherapy to target any remaining cancer cells. However, resistance to chemotherapy remains a significant challenge, contributing to recurrence and poor outcomes [14].

At the molecular level, OS is characterized by numerous genetic abnormalities, including chromosomal imbalances and mutations. Common alterations involve genes such as TP53, RB1, and MDM2, which play essential roles in cell cycle regulation and tumor suppression [15].

Several signaling pathways are implicated in osteosarcoma development and progression, including the p53 pathway, the Rb pathway, and the PI3K/AKT/mTOR pathway. Dysregulation of these pathways contributes to uncontrolled cell proliferation and survival [16].

The tumor microenvironment (TME) plays a significant role in osteosarcoma biology. Interactions between tumor cells and the surrounding stromal cells, extracellular matrix, and immune cells can influence tumor growth, metastasis, and treatment response [17].

Recent studies have highlighted the importance of epithelial–mesenchymal transition and cancer stem cell properties in osteosarcoma. EMT promotes invasive characteristics and may contribute to therapeutic resistance, while cancer stem cells are believed to be responsible for tumor recurrence [18]. Specific biomarkers, such as osteopontin and alkaline phosphatase, have been investigated for their prognostic value in osteosarcoma [19]. Identifying molecular markers can aid in risk stratification and the development of targeted therapies.

This narrative review aims to provide a comprehensive overview of the molecular mechanisms driving EMT in osteosarcoma, highlighting the involvement of signaling pathways such as TGF-β, transcription factors like Snail, Twist, and Zeb, and the role of microRNAs in modulating EMT. Furthermore, we discuss how EMT correlates with poor prognosis and therapy resistance in osteosarcoma patients, emphasizing the potential of targeting EMT for therapeutic intervention.

## 2. The Role of EMT in Cancer Progression

Epithelial–mesenchymal transition (EMT) is a pivotal biological process contributing to cancer progression by facilitating the transformation of epithelial cells into a mesenchymal phenotype [20]. This transition is characterized by the loss of cell–cell adhesion, primarily through the downregulation of epithelial markers such as E-cadherin, and the acquisition of migratory and invasive capabilities via the upregulation of mesenchymal markers like N-cadherin and vimentin. These changes enable cancer cells to detach from the primary tumor, invade surrounding tissues, and migrate through the extracellular matrix (ECM), ultimately leading to metastasis [21,22].

The significance of EMT in cancer lies in its multifaceted role in enhancing tumor aggressiveness. By promoting detachment and invasion, EMT enables cancer cells to penetrate the bloodstream or lymphatic system, facilitating the dissemination to distant sites [23]. Additionally, cells undergoing EMT exhibit increased resistance to apoptosis and conventional therapies, such as chemotherapy and radiotherapy. This resistance is often linked to the altered signaling pathways and microenvironmental interactions that protect tumor cells from therapeutic-induced cell death [24].

EMT is driven by several key signaling pathways, including the TGF-β pathway, which is one of the most potent inducers of EMT. TGF-β activates both Smad-dependent and non-Smad pathways, leading to the suppression of epithelial markers and the activation of mesenchymal genes [20,25]. Upon binding to its receptors, TGF-β activates intracellular signaling cascades primarily through the phosphorylation of Smad2 and Smad3 proteins. These phosphorylated Smads form a complex with Smad4, which translocates to the nucleus to regulate the expression of target genes involved in EMT, including key transcription factors such as Snail, Twist, and ZEB [26,27,28]. These transcription factors are instrumental in downregulating epithelial markers like E-cadherin while upregulating mesenchymal markers such as vimentin and fibronectin, thereby promoting the mesenchymal phenotype [29,30,31]. In addition to the canonical Smad pathway, TGF-β signaling also interacts with non-Smad pathways, including the Wnt/β-catenin and PI3K/AKT pathways, which further modulate the EMT process [32]. For instance, β-catenin can associate with phosphorylated Smad3, enhancing the transcriptional activity of EMT-related genes [33]. Moreover, TGF-β has been shown to induce the expression of matrix metalloproteinases (MMPs), which facilitate extracellular matrix remodeling and contribute to the invasive capabilities of cancer cells [27,29]. The Wnt/β-catenin and Notch signaling pathways also play crucial roles in maintaining the mesenchymal phenotype and promoting tumor cell migration and invasion. These pathways are often aberrantly activated in cancer, contributing to both local tumor invasion and metastatic spread [24].

A critical aspect of EMT is its regulation by transcription factors such as Snail, Slug, Twist, and ZEB1/2 [34,35,36]. These factors orchestrate the repression of epithelial characteristics and the activation of mesenchymal traits. For instance, Snail and ZEB1 downregulate E-cadherin, weakening cell adhesion, while promoting the expression of N-cadherin and vimentin, which enhance motility and invasive behavior [37]. In addition, Snail and Slug are known to repress E-cadherin, an essential protein for maintaining epithelial integrity, thereby facilitating the transition to a mesenchymal phenotype [38]. ZEB1, another critical player, has been shown to cooperate with Snail and Twist to enhance the repression of epithelial markers and promote the expression of mesenchymal markers [39,40]. The interplay between these TFs is complex; for example, ZEB1 can also be regulated by other factors such as Grainyhead-like 2 (GRHL2), which acts as a repressor of ZEB1, creating a feedback loop that fine-tunes the EMT process [39]. Moreover, the regulation of these transcription factors is influenced by post-translational modifications such as phosphorylation and ubiquitination. These modifications can alter the stability and activity of TFs, thereby impacting their ability to regulate EMT-related genes. For instance, phosphorylation of Snail can enhance its repressive activity on E-cadherin, while ubiquitination often leads to its degradation, thus modulating the EMT process [41]. The dynamic regulation of these TFs through PTMs underscores the complexity of the EMT regulatory network. In addition to the aforementioned transcription factors, other factors such as FOXC1 and SOX4 have been implicated in EMT regulation. FOXC1 has been shown to activate SNAI1, further promoting the mesenchymal phenotype in various cancer types [42]. Similarly, SOX4 has been identified as a master regulator of EMT, influencing the expression of genes involved in epigenetic reprogramming and cellular plasticity [43]. The involvement of these additional TFs highlights the multifaceted nature of EMT regulation and the potential for cross-talk between different signaling pathways. Furthermore, the context in which EMT occurs can significantly influence the roles of these transcription factors. For example, in the tumor microenvironment, the presence of inflammatory cytokines and growth factors can modulate the expression and activity of EMT-inducing TFs, thereby affecting tumor progression and metastasis [44,45]. The intricate balance between these factors and their regulatory networks is critical for understanding the mechanisms underlying EMT and its implications in cancer biology.

MicroRNAs (miRNAs) are small non-coding RNA molecules that play a significant role in the regulation of gene expression, particularly in the context of the epithelial-to-mesenchymal transition (EMT) [46]. One of the most studied miRNA families in relation to EMT is the miR-200 family, which includes miR-200a, miR-200b, miR-200c, miR-429, and miR-141. These miRNAs are known to suppress the expression of ZEB1 and ZEB2, transcription factors that promote EMT by repressing E-cadherin, a key epithelial marker [47,48]. The loss of miR-200 expression has been linked to increased tumor aggressiveness and metastasis [49]. Conversely, miR-21 has been implicated in promoting EMT by modulating tumor suppressor genes and enhancing cell invasion [50]. The downregulation of the miR-200 family is often associated with increased EMT and cancer progression, as seen in various cancer types, including ovarian and lung cancers [48,51]. For instance, loss of miR-200c expression has been linked to an aggressive, invasive phenotype in non-small cell lung cancer, highlighting its role as a tumor suppressor in the context of EMT [48]. In addition to the miR-200 family, other miRNAs such as miR-34 and miR-21 have been implicated in regulating EMT. miR-34 has been shown to form a feedback loop with SNAIL, a transcription factor that promotes EMT, thereby influencing the transition between epithelial and mesenchymal states [52]. Similarly, miR-21 has been reported to promote EMT by targeting various genes involved in the regulation of cell adhesion and migration, thus facilitating cancer cell invasion [53]. Moreover, miRNAs can also be influenced by external factors such as growth factors and cytokines. For example, TGF-β, a potent inducer of EMT, can modulate the expression of specific miRNAs, which in turn regulate the EMT process [54]. The interplay between TGF-β signaling and miRNA expression underscores the complexity of the regulatory networks involved in EMT. In gastric carcinoma, miR-223 has been shown to inhibit EMT by targeting Sp1, further illustrating the role of miRNAs in this process [55].

The tumor microenvironment plays a critical role in modulating EMT. Interactions with stromal cells, immune cells, and ECM components provide the necessary signals for tumor cells to undergo EMT. Cytokines such as TGF-β, IL-6, and IL-8 promote EMT by activating associated signaling pathways. Moreover, tumor cells secrete matrix metalloproteinases (MMPs), which degrade ECM components, facilitating invasion and remodeling the tumor microenvironment to support further tumor progression and metastasis [56,57].

One of the most critical consequences of EMT is its role in metastasis, the process by which cancer cells spread from the primary tumor to distant organs. Cells that have undergone EMT are better equipped to survive the hostile conditions of the circulatory system, displaying increased resistance to stress and apoptosis [49]. Upon reaching distant tissues, cancer cells can undergo a reverse process known as mesenchymal–epithelial transition (MET), which allows them to regain epithelial characteristics and establish secondary tumors. The ability of cancer cells to switch between EMT and MET is vital for successful metastasis, as it enables them to adapt to different microenvironments and colonize new tissues [58].

EMT is also closely linked to cancer stem cell (CSC) properties, which contribute to tumor heterogeneity, therapy resistance, and recurrence. Cells undergoing EMT often acquire stem-like characteristics, such as self-renewal and enhanced tumor-initiating capabilities. These CSC-like cells are more resistant to treatment and are thought to play a central role in tumor relapse following therapy. The presence of CSCs in tumors complicates treatment, as they contribute to a more aggressive and resilient tumor population [49,59].

Clinically, EMT presents a significant challenge due to its association with therapy resistance. Cells that have undergone EMT are less susceptible to apoptosis and tend to survive conventional treatments [60]. This resistance is mediated by altered signaling pathways and protective interactions within the tumor microenvironment, which shield tumor cells from the cytotoxic effects of treatment. As such, targeting the pathways involved in EMT, such as TGF-β, Wnt/β-catenin, or Notch, holds promise for novel therapeutic strategies aimed at reducing metastasis and overcoming therapy resistance.

In conclusion, EMT is a central process in cancer progression, contributing to tumor invasion, metastasis, and resistance to therapy. Understanding the complex molecular mechanisms governing EMT is crucial for developing innovative therapies that target this process, with the ultimate goal of improving clinical outcomes for cancer patients. By inhibiting EMT or reversing its effects, we may enhance treatment responses, reduce metastatic spread, and improve the overall prognosis for patients with cancer.

## 3. Epithelial–Mesenchymal Transition in Osteosarcoma: Evidence from Preclinical and Clinical Studies

Epithelial–mesenchymal transition (EMT) plays a pivotal role in the progression of osteosarcoma, influencing its invasiveness, metastatic potential, and response to treatment [61]. Preclinical studies utilizing various experimental models shed light on the molecular mechanisms driving EMT, providing insights that may lead to innovative therapeutic interventions. These models include established osteosarcoma cell lines such as U2OS, MG-63, HOS, and SaOS-2, which allow researchers to explore the changes associated with EMT in a controlled environment [62,63]. Additionally, xenograft models, where osteosarcoma cells are implanted into immunocompromised mice, enable the investigation of tumor behavior in a living organism and the effects of potential treatments on EMT [64].

Key molecular mechanisms regulating EMT in osteosarcoma have been identified, including TGF-β signaling, which is a primary inducer of EMT. Activation of TGF-β in osteosarcoma cells leads to decreased expression of E-cadherin and increased expression of mesenchymal markers such as N-cadherin and vimentin, thereby enhancing invasive and metastatic capabilities [65]. The Wnt/β-catenin pathway is another crucial player, with studies indicating that stabilized β-catenin accumulates in the nucleus and regulates genes associated with mesenchymal transition [66]. Other growth factors and cytokines, including epidermal growth factor (EGF) [67] and interleukin-6 (IL-6) [68], have also been implicated in promoting EMT in osteosarcoma, contributing to enhanced tumor invasion.

EMT significantly impacts the invasiveness and metastatic potential of osteosarcoma [61]. Studies demonstrate that osteosarcoma cells undergoing EMT possess an increased capacity to invade the extracellular matrix (ECM), with in vitro experiments showing that cells with elevated mesenchymal marker expression migrate more efficiently through collagen matrices than those maintaining an epithelial phenotype. Furthermore, animal models of metastatic osteosarcoma indicate that EMT is essential for the formation of metastases in distal organs, such as the lungs, where cells that have undergone EMT exhibit enhanced survival in circulation and a greater ability to colonize new tissues [69,70].

Therapeutic interventions targeting EMT are being actively explored. Preclinical studies are investigating the use of inhibitors that target signaling pathways associated with EMT as adjunctive treatments for osteosarcoma. For instance, inhibiting TGF-β or the Wnt/β-catenin pathway may reduce the invasion and metastasis of the tumor [71,72,73,74]. Combination therapies are also being considered, as evidence suggests that pairing chemotherapy with agents that block EMT may improve therapeutic responses and decrease recurrence rates, given that mesenchymal phenotype cells often exhibit greater resistance to standard treatments [75,76].

The identification of EMT biomarkers is crucial for understanding osteosarcoma biology and its progression. Key biomarkers associated with EMT include E-cadherin, N-cadherin, vimentin, and fibronectin. E-cadherin, a cell–cell adhesion molecule, is typically downregulated during EMT, and reduced levels correlate with increased invasiveness and metastatic potential [77]. Conversely, the upregulation of N-cadherin, which enhances cell motility, and vimentin, a marker of mesenchymal transition, signifies a more aggressive tumor phenotype [78]. Fibronectin, an ECM protein, is also upregulated during EMT and facilitates tumor invasion [79]. Understanding the expression of these biomarkers not only aids in prognosis but also opens avenues for targeted therapies aimed at reversing EMT or inhibiting its associated signaling pathways.

The clinical implications of EMT biomarkers are profound, influencing therapeutic targeting, monitoring treatment responses, and facilitating personalized medicine approaches in osteosarcoma. The presence of EMT features can affect the efficacy of conventional and targeted therapies, highlighting the need for clinicians to consider a tumor’s EMT status when selecting treatment strategies [80]. By identifying patients with specific EMT-related characteristics, clinicians can tailor treatment regimens to enhance efficacy and minimize resistance.

Monitoring the dynamics of EMT biomarkers during treatment provides valuable insights into treatment response. An increase in epithelial markers or a decrease in mesenchymal markers may signify a favorable response, while persistent or increased mesenchymal marker expression could indicate resistance and necessitate alternative treatment strategies [80]. Real-time assessments through liquid biopsies that monitor circulating tumor cells (CTCs) and their EMT status can further enhance clinical decision-making [81].

In conclusion, the evidence from preclinical and clinical studies highlights the critical role of EMT in osteosarcoma, influencing not only its biology but also its clinical outcomes. Understanding the mechanisms and implications of EMT will be essential for optimizing treatment strategies and improving patient care. Continued research into EMT and its biomarkers will pave the way for innovative therapies and personalized treatment approaches, ultimately enhancing survival rates and quality of life for osteosarcoma patients.

## 4. Molecular Mechanisms of EMT in Osteosarcoma

Epithelial–mesenchymal transition (EMT) plays a critical role in the development of osteosarcoma, influencing tumor invasion and metastasis [61]. This section explores the molecular pathways involved in EMT, focusing on the TGF-β signaling pathway, relevant transcription factors, and interactions with microRNAs and other post-transcriptional regulatory molecules.

### 4.1. The TGF-β Pathway in EMT and Osteosarcoma

The Transforming Growth Factor-beta (TGF-β) signaling pathway is a crucial regulator of various cellular processes, including cell proliferation, differentiation, and apoptosis [82]. In the context of EMT, TGF-β acts as a potent inducer, promoting the transition from an epithelial phenotype to a mesenchymal one [20,83]. There are three isoforms of TGF-β (TGF-β1, TGF-β2, and TGF-β3), with TGF-β1 being the most studied in cancer biology. Upon binding to its receptors (TGF-βRI and TGF-βRII), TGF-β activates downstream signaling pathways, predominantly the Smad-dependent and Smad-independent pathways [25,84].

TGF-β induces EMT through several mechanisms, including the inhibition of E-cadherin, a critical epithelial adhesion molecule, and the activation of mesenchymal markers such as N-cadherin and vimentin. This transition enhances cellular motility and invasiveness [20]. In osteosarcoma, TGF-β is secreted by various cells within the tumor microenvironment, influencing neighboring osteosarcoma cells to undergo EMT. The activation of the TGF-β pathway is linked to increased invasiveness and metastatic potential, contributing to the high incidence of lung metastases associated with this cancer [85,86].

Furthermore, the TGF-β pathway has been implicated in the development of resistance to chemotherapy. Tumors exhibiting high TGF-β activity may have a more aggressive phenotype, enabling them to evade the cytotoxic effects of conventional treatments, leading to treatment failure and disease recurrence [87,88]. Targeting the TGF-β pathway is being explored as a therapeutic strategy to counteract EMT and improve treatment outcomes in osteosarcoma. Several TGF-β inhibitors, such as small molecule inhibitors, monoclonal antibodies, and receptor blockers, are under investigation in preclinical and clinical settings [71,72,89]. Combining TGF-β inhibitors with standard chemotherapy or novel targeted therapies may enhance treatment efficacy, potentially reversing EMT characteristics and making tumor cells more susceptible to cytotoxic agents [75,76].

### 4.2. The Role of Transcription Factors in EMT and Osteosarcoma

Transcription factors are crucial regulators of the EMT process, controlling the expression of genes associated with cell adhesion, migration, and invasion. In osteosarcoma, key transcription factors such as Snail, Twist, and Zeb are instrumental in driving EMT and contributing to tumor progression [90].

#### 4.2.1. Snail

Snail is a zinc finger transcription factor that acts as a master regulator of EMT and is primarily involved in repressing the expression of E-cadherin. Snail binds to the E-box elements in the E-cadherin promoter, leading to its transcriptional repression [91]. This downregulation of E-cadherin is associated with increased cell motility and invasion, which are hallmarks of aggressive tumor behavior [92]. Studies have shown that silencing Snail expression in osteosarcoma cell lines results in the restoration of E-cadherin levels, thereby reducing cell migration and invasion [92].

Its upregulation is often triggered by TGF-β signaling, facilitating cell detachment and enhancing the migratory capacity of tumor cells [34]. Therefore, high Snail expression in osteosarcoma is associated with increased invasiveness and poorer prognosis, serving as a predictive biomarker for treatment response and patient outcomes [77,93]. In addition to repressing E-cadherin, Snail promotes the expression of mesenchymal markers such as N-cadherin, vimentin, and fibronectin. This shift in expression profile is indicative of the EMT process and contributes to the invasive characteristics of osteosarcoma cells. The upregulation of these markers is often correlated with poor prognosis in osteosarcoma patients [94,95]. Snail’s activity in osteosarcoma is modulated by various signaling pathways, including the NF-κB and STAT3 pathways. For instance, the NF-κB pathway has been shown to enhance Snail expression, thereby promoting EMT and tumor progression [68,92]. Additionally, the STAT3 signaling pathway can regulate Snail expression in response to inflammatory cytokines, further linking Snail to the tumor microenvironment and its effects on metastasis [68]. Snail does not act in isolation; it interacts with other transcription factors that regulate EMT. For example, it has been shown to cooperate with Twist and ZEB1 to reinforce the mesenchymal phenotype in osteosarcoma cells [67,96]. This cooperative interaction amplifies the EMT program, facilitating enhanced invasiveness and metastatic potential.

#### 4.2.2. Twist

Twist is another key transcription factor that promotes EMT by regulating the expression of several genes involved in the transition process. Like Snail, Twist inhibits E-cadherin expression by binding to its promoter, while also promoting the expression of mesenchymal markers such as N-cadherin and vimentin [97]. The activity of Twist in osteosarcoma is influenced by various signaling pathways, including the TGF-β and PI3K/AKT pathways. For instance, TGF-β signaling has been shown to enhance Twist expression, thereby facilitating EMT and tumor progression. Additionally, the PI3K/AKT pathway can modulate Twist expression in response to different stimuli, further linking Twist to the tumor microenvironment and its effects on metastasis [98]. Increased Twist expression has been linked to advanced disease stages and poorer overall survival in osteosarcoma patients, suggesting that targeting Twist may provide therapeutic opportunities to reverse EMT and enhance sensitivity to chemotherapy [90,99].

#### 4.2.3. Zeb (Zinc Finger E-Box Binding Homeobox)

Zeb is a family of transcription factors, including Zeb1 and Zeb2, which are critical regulators of EMT. They function by repressing epithelial markers while promoting mesenchymal characteristics. Zeb1 and Zeb2 act as transcriptional repressors of E-cadherin. Loss of E-cadherin leads to decreased cell–cell adhesion, facilitating the detachment and dissemination of tumor cells [100]. They also upregulate N-cadherin and vimentin, enhancing the mesenchymal phenotype. This shift increases cell migration and invasiveness, which are key characteristics of metastatic osteosarcoma [100]. Elevated levels of Zeb1 have been correlated with metastasis and poor prognosis in various cancers, including osteosarcoma. Studies indicate that targeting Zeb could be an effective strategy to inhibit EMT and improve therapeutic responses [90,101].

#### 4.2.4. Interactions Between Transcription Factors

Snail, Twist, and Zeb often interact and cooperate to regulate the EMT process, creating a complex regulatory network that drives the transition from epithelial to mesenchymal phenotypes in osteosarcoma cells. Feedback mechanisms exist in which the expression of one transcription factor can influence the expression of others, enhancing the EMT process [90].

### 4.3. Interaction with MicroRNAs and Other Post-Transcriptional Regulatory Molecules

The regulation of EMT is also influenced by post-transcriptional regulatory molecules, including microRNAs (miRNAs), long non-coding RNAs (lncRNAs) and circular RNAs (circRNAs). These interactions play a vital role in modulating gene expression during EMT in osteosarcoma [102,103].

#### 4.3.1. MicroRNAs (miRNAs)

MicroRNAs are small, non-coding RNA molecules that regulate gene expression at the post-transcriptional level by binding to complementary sequences on target mRNA, leading to mRNA degradation or inhibition of translation. Various miRNAs are implicated in the regulation of EMT by targeting transcription factors and their downstream effectors [104,105]. For example, the miR-200 family (including miR-200a, miR-200b, and miR-200c) inhibits EMT by directly targeting Zeb1 and Zeb2, thereby promoting the expression of E-cadherin and maintaining the epithelial phenotype [106,107].

Conversely, miR-21 is often upregulated in osteosarcoma and has been associated with promoting EMT. It targets various tumor suppressor genes and enhances the expression of mesenchymal markers, contributing to increased cell migration and invasion [108]. Additionally, miR-34a, a direct target of Snail, inhibits EMT by downregulating Snail expression, but its expression is often decreased in osteosarcoma, allowing Snail levels to rise [109].

#### 4.3.2. Long Non-Coding RNAs (lncRNAs)

Long non-coding RNAs are another class of regulatory RNAs that can influence EMT. They may act as scaffolds for transcription factors, modulating their activity, or compete with miRNAs for binding to target mRNAs [110]. For instance, the lncRNA HOTAIR promotes EMT by silencing epithelial markers, while MALAT1 has been shown to modulate the expression of key EMT transcription factors, with its overexpression in osteosarcoma linked to increased invasiveness and metastasis [111].

#### 4.3.3. Feedback Loops and Regulatory Networks

The interactions between transcription factors and miRNAs/lncRNAs create complex regulatory networks that finely tune the EMT process. For example, while transcription factors like Snail and Zeb can downregulate miR-200 family members, those miRNAs can, in turn, inhibit the expression of the same transcription factors [112,113]. Understanding these dynamic interactions is crucial for identifying potential therapeutic targets and improving treatment strategies in osteosarcoma.

#### 4.3.4. Conclusion

The interplay between transcription factors and post-transcriptional regulatory molecules, such as microRNAs and long non-coding RNAs, is vital in modulating EMT in osteosarcoma. These regulatory interactions contribute to the complex signaling networks that govern tumor progression, invasiveness, and metastasis. Further research into these interactions may reveal novel therapeutic strategies for targeting EMT and improving outcomes in osteosarcoma patients.

## 5. EMT and Therapy Resistance in Osteosarcoma

### 5.1. EMT and Chemoresistance

Epithelial–mesenchymal transition (EMT) is a crucial biological process that plays a significant role in the progression and metastasis of various cancers, including osteosarcoma [114]. One of the critical implications of EMT is its association with chemoresistance, which presents a major challenge in effectively treating osteosarcoma [115]. Understanding the mechanisms by which EMT contributes to chemoresistance is essential for developing more effective therapeutic strategies aimed at improving patient outcomes.

Cells undergoing EMT typically exhibit alterations in their phenotype, leading to changes in drug sensitivity. Mesenchymal cells often show increased expression of ATP-binding cassette (ABC) transporters, such as P-glycoprotein (MDR1), which actively expel chemotherapeutic agents from the cells, thereby reducing drug efficacy [116,117,118]. Additionally, EMT is linked to the upregulation of anti-apoptotic proteins like Bcl-2 and survivin, along with the downregulation of pro-apoptotic factors such as Bax. This imbalance allows mesenchymal cells to evade the apoptotic signals that would normally induce cell death in epithelial cells, thus enabling them to survive in the presence of chemotherapeutic agents [119].

EMT also activates various survival signaling pathways, such as the PI3K/Akt and MAPK pathways, which enhance cell survival and proliferation. These pathways contribute to the resistance of osteosarcoma cells to chemotherapeutic agents by increasing their resilience to drug-induced stress [120]. Consequently, patients with osteosarcoma characterized by EMT often face poorer treatment outcomes, as the presence of mesenchymal markers like N-cadherin and vimentin has been associated with lower overall survival rates and higher recurrence following chemotherapy [121]. Moreover, tumors exhibiting EMT tend to be less responsive to conventional chemotherapeutic agents, such as doxorubicin and cisplatin, commonly used in osteosarcoma treatment. This resistance complicates treatment regimens and facilitates disease progression [80,122].

To overcome chemoresistance associated with EMT, several strategies can be employed. Targeting the molecular pathways driving EMT may enhance chemosensitivity; for instance, inhibiting TGF-β signaling can prevent EMT and reverse the associated resistance, thereby making tumor cells more susceptible to chemotherapy [123]. Additionally, combining traditional chemotherapy with agents that inhibit EMT or reverse mesenchymal characteristics could significantly improve treatment efficacy. For example, utilizing miRNA mimics, such as those from the miR-200 family, which suppress EMT, can enhance the effectiveness of existing therapies [124]. Furthermore, the use of nanoparticles to deliver chemotherapeutic agents directly to mesenchymal cells may bypass some of the resistance mechanisms. Nanoparticle formulations can also improve drug solubility and stability, ultimately leading to better therapeutic outcomes [125,126].

### 5.2. New Therapeutic Directions Targeting EMT

Epithelial–mesenchymal transition (EMT) is a crucial process in the progression and metastasis of osteosarcoma, leading to treatment resistance and poor prognosis. Targeting the molecular mechanisms underlying EMT offers promising therapeutic strategies [114].

Inhibitors of the TGF-β signaling pathway, such as SB431542 and neutralizing antibodies, can prevent EMT induction and enhance sensitivity to chemotherapy [127]. Additionally, inhibiting the Wnt/β-catenin pathway with agents like XAV939 can reverse EMT and reduce invasiveness [128].

One of the key factors involved in EMT is the transcription factor DEC1, which has been shown to upregulate mesenchymal markers such as N-cadherin and vimentin while downregulating the epithelial marker E-cadherin [26]. This suggests that DEC1 plays a significant role in promoting the aggressive characteristics of osteosarcoma cells. By inhibiting DEC1 or its downstream effects, it may be possible to reverse EMT and enhance the sensitivity of osteosarcoma cells to conventional therapies. Additionally, the Notch signaling pathway has been implicated in EMT and chemoresistance in osteosarcoma. Shiota et al. (2012) demonstrated that low concentrations of doxorubicin could induce EMT through the activation of Notch signaling [27]. Understanding the mechanisms by which Notch signaling contributes to EMT may lead to the development of novel therapeutic strategies aimed at overcoming chemoresistance and improving treatment outcomes.

Fibulin-4 is another important player in the EMT process in osteosarcoma. Suzuki et al. (2019) found that fibulin-4 promotes invasion and metastasis by inducing EMT via the PI3K/Akt/mTOR signaling pathway. Targeting fibulin-4 or its associated pathways may provide a new avenue for therapeutic intervention, potentially reducing the invasive capabilities of osteosarcoma cells [28].

Agents targeting the PI3K/Akt/mTOR pathway, such as rapamycin and everolimus, disrupt survival signals linked to EMT, improving chemotherapy responsiveness [129,130]. Furthermore, the PI3K/AKT/mTOR signaling pathway has been shown to play a significant role in EMT and cancer progression [131]. Kita et al. (2017) reported that STEAP2 promotes osteosarcoma progression by inducing EMT via the PI3K/AKT/mTOR pathway. Targeting this pathway may not only inhibit EMT but also enhance the response to immunotherapy by modulating the tumor microenvironment [29].

Direct inhibition of EMT transcription factors like Snail, Twist, and Zeb can restore the epithelial phenotype in osteosarcoma cells [90].

Small molecules or RNA interference strategies can effectively block these transcription factors. MicroRNA-based approaches, particularly the restoration of miR-200 family expression, can inhibit Zeb and promote E-cadherin re-expression, reversing EMT [106,107].

Moreover, the use of compounds such as nitidine chloride has shown promise in suppressing EMT in osteosarcoma. Kita et al. (2017) reported that nitidine chloride inhibits cell migration and invasion through the Akt/GSK-3β/Snail signaling pathway, highlighting the potential of repurposing existing drugs to target EMT in osteosarcoma [29]. Cyr61, a cysteine-rich protein, has also been identified as a promoter of EMT and tumor metastasis in osteosarcoma. Park et al. (2011) demonstrated that Cyr61 enhances EMT through the Raf-1/MEK/ERK/Elk-1/TWIST-1 signaling pathway. The knockdown of Cyr61 resulted in decreased invasion and migration of osteosarcoma cells, suggesting that it may serve as a novel therapeutic target [30]. Long non-coding RNAs (lncRNAs) have emerged as important regulators of EMT in various cancers, including osteosarcoma. For instance, Sung et al. (2014) identified that the lncRNA BCRT1 facilitates osteosarcoma progression by regulating the miR-1303/FGF7 axis, which is involved in EMT [31]. Targeting lncRNAs that promote EMT could provide a new strategy for inhibiting tumor progression.

Combining traditional chemotherapy with EMT inhibitors may enhance therapeutic efficacy. For example, pairing doxorubicin with TGF-β inhibitors can reverse chemoresistance [132]. Additionally, integrating immunotherapy with EMT-targeting agents can improve immune responses by addressing immune evasion linked to EMT [133].

Nanotechnology offers innovative drug delivery solutions. Nanoparticles can deliver EMT inhibitors directly to tumor cells, enhancing local drug concentrations while minimizing systemic toxicity [134]. Targeted delivery systems can also selectively target EMT-expressing cells, improving therapeutic specificity.

Continued clinical evaluation of EMT-targeted therapies and the identification of biomarkers associated with EMT will be essential for patient stratification and improving treatment outcomes. Ongoing clinical trials are crucial to assess the safety and efficacy of these innovative strategies in osteosarcoma.

### 5.3. EMT as a Target for Immunotherapy

EMT significantly influences the progression of osteosarcoma, contributing to tumor heterogeneity and resistance to conventional therapies. Targeting EMT within the context of immunotherapy offers a novel approach to enhance anti-tumor immune responses [135,136].

Understanding the role of EMT in tumor immunology reveals that the EMT process is associated with an immunosuppressive microenvironment. Mesenchymal-like cancer cells often express immune checkpoint molecules such as PD-L1, which inhibit T cell activation and promote immune evasion [137,138]. PD-L1 is a pivotal player in the biology of osteosarcoma, influencing tumor growth, metastasis, and immune evasion. Its expression is associated with poor prognosis and drug resistance, making it a promising target for immunotherapy. Studies have shown that osteosarcoma cells undergoing EMT often express higher levels of PD-L1, which interacts with PD-1 on T cells, leading to the inhibition of T cell activation and promoting an immunosuppressive microenvironment [26]. This interaction allows tumor cells to evade immune surveillance, thereby facilitating tumor growth and metastasis. Targeting the EMT process may enhance the effectiveness of immune checkpoint inhibitors. For instance, Fei et al. (2019) demonstrated that inhibiting the Wnt–Axin2–Snail signaling cascade could reduce PD-L1 expression in osteosarcoma cells, suggesting that the modulation of EMT pathways may sensitize tumors to immunotherapy [26]. By disrupting the signaling pathways that promote EMT, it may be possible to restore T cell activity and improve the efficacy of existing immunotherapeutic strategies. One of the key findings regarding PD-L1 in osteosarcoma is its association with drug resistance. Fei et al. (2019) [26] demonstrated that the overexpression of PD-L1 in osteosarcoma cells was linked to increased resistance against cisplatin, a common chemotherapeutic agent used in treatment. The study highlighted that exosomal PD-L1 not only contributed to lung metastasis but also served as a potential biomarker for disease progression. This suggests that targeting PD-L1 may enhance the efficacy of existing therapies by overcoming resistance mechanisms. Moreover, PD-L1 expression has been shown to facilitate osteosarcoma cell proliferation. Shiota et al. (2012) [27] reported that co-culturing PD-1-overexpressing lymphocytes with PD-L1-overexpressing osteosarcoma cells resulted in enhanced tumor cell growth. The study also indicated that the combination of anti-PD-1 antibodies with cisplatin could promote apoptosis in osteosarcoma cells, suggesting a synergistic effect that could be exploited for therapeutic benefit. The role of PD-L1 in osteosarcoma is further underscored by its involvement in the metastatic process. Suzuki et al. (2019) identified the LINC00657/miR-106a/PD-L1 axis as a promoter of osteosarcoma metastasis, indicating that PD-L1 plays a significant role in the dissemination of cancer cells [28]. Additionally, the expression of PD-L1 has been correlated with the presence of tumor-infiltrating lymphocytes (TILs), which are critical for anti-tumor immunity. Kita et al. (2017) demonstrated that blockade of the PD-1/PD-L1 interaction could enhance T cell immunity against osteosarcoma, providing a rationale for immunotherapeutic approaches [29].

Targeting key signaling pathways involved in EMT can enhance the susceptibility of tumor cells to immune-mediated killing. For instance, inhibitors of TGF-β and Wnt/β-catenin pathways can reverse EMT and reinvigorate anti-tumor immunity [139].

Combining EMT inhibitors with immune checkpoint inhibitors, such as anti-PD-1 and anti-CTLA-4, may enhance therapeutic efficacy by promoting T cell activation [140,141]. Restoring epithelial characteristics through EMT reversal can also improve antigen presentation to immune cells, facilitating better recognition and targeting of tumor cells.

Developing therapeutic vaccines targeting specific antigens associated with EMT can enhance immune responses against osteosarcoma. Personalized vaccines utilizing neoantigens derived from individual tumors may allow for tailored immune responses [85].

Clinical trials focusing on the combined targeting of EMT and immunotherapy are essential to evaluate the safety and efficacy of these strategies in osteosarcoma. Identifying specific biomarkers associated with EMT and immune evasion will assist in selecting patients most likely to benefit from these therapies.

## 6. Therapeutic Perspectives and Future Directions

### 6.1. Utilizing EMT Biomarkers for Personalized Therapy in Osteosarcoma

EMT is a critical process in osteosarcoma progression, and biomarkers associated with EMT can provide insights into tumor behavior and treatment responses. Identifying these biomarkers, such as E-cadherin, which is a hallmark of EMT, can indicate tumor aggressiveness. Low levels of E-cadherin may suggest that patients could benefit from therapies targeting EMT pathways. High expression of vimentin, a mesenchymal marker, is linked to increased invasiveness, while the expression levels of transcription factors Snail, Twist, and Zeb can help determine EMT status and prognosis. Additionally, specific microRNAs, particularly from the miR-200 family, can serve as indicators of the EMT phenotype and predict therapeutic responses.

Personalized treatment strategies can be developed based on these biomarkers. For instance, patients exhibiting high TGF-β signaling or low E-cadherin levels may respond well to TGF-β inhibitors.

Recent studies have demonstrated that inhibiting the TGF-β signaling pathway can significantly impact osteosarcoma cell behavior. For instance, Fei et al. (2019) investigated the role of the TGF-β/Smad3 signaling cascade in osteosarcoma cells and found that the ALK5 inhibitor SD208 effectively blocked TGF-β-induced transcriptional activation [26]. This inhibition resulted in reduced metastatic potential, highlighting the therapeutic promise of TGF-β inhibitors in managing osteosarcoma. Moreover, the role of TGF-β in promoting heterogeneity and drug resistance has been well documented. Shiota et al. (2012) reported that TGF-β signaling contributes to the development of drug-resistant cancer cell populations [27]. This finding suggests that TGF-β inhibitors could be used in combination with conventional chemotherapy to enhance treatment efficacy and overcome resistance mechanisms. In addition to direct effects on tumor cells, TGF-β signaling influences the tumor microenvironment, which is crucial for osteosarcoma progression. Suzuki et al. (2019) demonstrated that miRNA-21 inhibition could suppress osteosarcoma cell proliferation by targeting the TGF-β1 signaling pathway [28]. This indicates that TGF-β inhibitors may also modulate the tumor microenvironment, potentially enhancing immune responses against osteosarcoma.

Despite the promising findings from in vitro and preclinical studies, it is important to note that most current research on TGF-β inhibitors in osteosarcoma is based on laboratory models. The translation of these findings into clinical settings poses significant challenges. For instance, the complexity of TGF-β signaling and its context-dependent effects may lead to variable responses in patients. Additionally, potential side effects associated with systemic TGF-β inhibition must be carefully considered. Future research should focus on elucidating the specific mechanisms by which TGF-β inhibitors exert their effects in osteosarcoma, as well as identifying biomarkers that predict patient responses to these therapies. Furthermore, clinical trials are needed to evaluate the safety and efficacy of TGF-β inhibitors in combination with existing treatment modalities, such as chemotherapy and immunotherapy.

Combination therapies can also be tailored according to biomarker profiles, such as combining EMT inhibitors with traditional chemotherapy for patients with high vimentin expression. Regular monitoring of these biomarkers during treatment can help adjust therapy as needed, maximizing effectiveness.

EMT biomarkers also have prognostic and predictive value, aiding in patient stratification and informing treatment intensity. Incorporating EMT biomarker assessments into clinical trials can help identify responsive populations and validate novel therapies. Future research should focus on discovering new biomarkers and developing comprehensive profiles to better personalize treatment strategies.

In summary, leveraging EMT biomarkers for personalized therapy in osteosarcoma can enhance treatment efficacy and improve patient outcomes by enabling tailored therapeutic approaches that reflect the unique characteristics of each tumor.

### 6.2. Future Research Needed for a Better Understanding of EMT in Osteosarcoma

To advance the understanding of EMT in osteosarcoma and develop effective therapeutic strategies, several key research directions must be pursued. Mechanistic studies are essential to elucidate the intricate signaling pathways regulating EMT, including the interplay between growth factors, cytokines, and transcription factors [142]. Additionally, examining how the tumor microenvironment influences EMT through interactions with stromal components, immune cells, and fibroblasts is crucial [143].

One promising avenue for future research is the exploration of long non-coding RNAs (lncRNAs) and their roles in regulating EMT. For instance, Fei et al. (2019) demonstrated that lncRNA BCRT1 facilitates osteosarcoma progression by modulating the miR-1303/FGF7 axis, which is involved in EMT [26]. Understanding the specific mechanisms by which lncRNAs influence EMT could reveal novel therapeutic targets and biomarkers for osteosarcoma. Additionally, microRNAs (miRNAs) have emerged as critical regulators of EMT in various cancers, including osteosarcoma. Shiota et al. (2012) found that miR-19 promotes cell proliferation, invasion, and migration by inhibiting SPRED2-mediated autophagy in osteosarcoma cells [27]. Investigating the roles of different miRNAs in EMT could provide insights into their potential as therapeutic targets or prognostic markers. The involvement of signaling pathways in EMT also requires further exploration. Suzuki et al. (2019) highlighted the significance of the TGF-β-induced EMT signaling pathway in osteosarcoma progression [28]. Future studies should focus on dissecting the intricate signaling networks that regulate EMT, including the interplay between the TGF-β, Notch, and Wnt pathways, to identify potential points of intervention. Moreover, the relationship between EMT and drug resistance in osteosarcoma is an area ripe for investigation. Kita et al. (2017) reported that cisplatin treatment promotes mesenchymal-like characteristics in osteosarcoma cells through the activation of Snail [29]. The role of cancer stem cells (CSCs) in EMT and osteosarcoma progression is another critical area for future research. Yu et al. and Park et al. (2011) noted that CSCs are involved in the initiation and progression of malignant tumors, including osteosarcoma [30]. Investigating the relationship between CSCs and EMT could provide insights into tumor heterogeneity and the mechanisms underlying metastasis and recurrence. Additionally, the tumor microenvironment plays a significant role in regulating EMT and immune responses. Sung et al. (2014) emphasized the importance of the NF-κB pathway in mediating EMT in osteosarcoma [31]. Future studies should explore how the tumor microenvironment influences EMT and immune evasion, potentially leading to novel immunotherapeutic strategies. Finally, the development of novel therapeutic agents targeting EMT is essential to improve osteosarcoma treatment outcomes. Yang et al. (2022) demonstrated that the lncRNA SNHG16 promotes EMT by upregulating ITGA6 through miR-488 inhibition [32]. Identifying and characterizing new compounds that can effectively inhibit EMT could provide additional treatment options for patients with osteosarcoma.

Biomarker discovery and validation should focus on identifying novel biomarkers associated with EMT, including microRNAs, proteins, and genetic signatures that correlate with EMT status and clinical outcomes. Large-scale studies are necessary to validate these biomarkers in clinical samples and establish their prognostic and predictive value.

Understanding tumor heterogeneity is also vital. Research should investigate the plasticity of osteosarcoma cells undergoing EMT, focusing on how different subpopulations transition between epithelial and mesenchymal states. Utilizing advanced techniques like single-cell RNA sequencing can help characterize EMT heterogeneity and reveal distinct cellular populations involved in tumor progression.

Investigating therapeutic targeting of EMT through advanced preclinical models, such as patient-derived xenografts and organoids, will provide valuable data on treatment efficacy. Identifying optimal combinations of EMT inhibitors with existing therapies can also enhance treatment effectiveness.

Future clinical trials should incorporate assessments of EMT biomarkers to tailor treatments and improve the likelihood of therapeutic success. Longitudinal studies can monitor EMT status during treatment to understand its dynamic nature and implications for resistance and recurrence.

Lastly, research should explore how EMT contributes to the metastatic spread of osteosarcoma, identifying molecular mechanisms involved and targeting metastatic niches influenced by the microenvironment.

In conclusion, addressing these research areas is vital for improving the understanding of EMT in osteosarcoma, paving the way for personalized treatments that enhance survival rates and quality of life for patients.

## 7. Conclusions

Epithelial–mesenchymal transition (EMT) is crucial in osteosarcoma progression, facilitating tumor invasion, metastasis, and chemoresistance. EMT-associated biomarkers such as E-cadherin, vimentin, Snail, and Twist are valuable for prognosis and treatment response, supporting personalized therapy approaches. Strategies to inhibit EMT through key signaling pathways (TGF-β, Wnt) and transcription factors show promise in enhancing treatment efficacy when combined with conventional therapies. Further investigation is essential to elucidate the molecular mechanisms of EMT, identify novel biomarkers, and validate their clinical relevance. Focus on the therapeutic potential of EMT modulation, longitudinal studies to track EMT status during treatment, and integrating biomarker assessments into clinical trials will advance personalized therapy in osteosarcoma. In addition, incorporating EMT research findings into clinical practice can improve prognostic accuracy and tailor treatment strategies, ultimately enhancing patient outcomes and survival rates.
